# Magnetic Resonance Imaging Studies of Postpartum Depression: An Overview

**DOI:** 10.1155/2015/913843

**Published:** 2015-08-11

**Authors:** Marco Fiorelli, Franca Aceti, Isabella Marini, Nicoletta Giacchetti, Enrica Macci, Emanuele Tinelli, Valentina Calistri, Valentina Meuti, Francesca Caramia, Massimo Biondi

**Affiliations:** Department of Neurology and Psychiatry, Sapienza University of Rome, Viale dell'Università 30, 00185 Rome, Italy

## Abstract

Postpartum depression is a frequent and disabling condition whose pathophysiology is still unclear. In recent years, the study of the neural correlates of mental disorders has been increasingly approached using magnetic resonance techniques. In this review we synthesize the results from studies on postpartum depression in the context of structural, functional, and spectroscopic magnetic resonance studies of major depression as a whole. Compared to the relative wealth of data available for major depression, magnetic resonance studies of postpartum depression are limited in number and design. A systematic literature search yielded only eleven studies conducted on about one hundred mothers with postpartum depression overall. Brain magnetic resonance findings in postpartum depression appear to replicate those obtained in major depression, with minor deviations that are not sufficient to delineate a distinct neurobiological profile for this condition, due to the small samples used and the lack of direct comparisons with subjects with major depression. However, it seems reasonable to expect that studies conducted in larger populations, and using a larger variety of brain magnetic resonance techniques than has been done so far, might allow for the identification of neuroimaging signatures for postpartum depression.

## 1. Introduction

Postpartum depression (PPD) is a common disease that affects approximately 10–20% of new mothers in the western world [1]; it is deemed a public health problem [2]. It is important to emphasise that while PPD may appear de novo, in most cases it evolves from inadequately recognised prenatal depression, which therefore remains undiagnosed and untreated during pregnancy [3]. Depression during pregnancy is associated with an increased risk of an operative delivery and preeclampsia [4]. An unrecognised and untreated depression may cause mothers to neglect themselves; they may also pay little attention to obstetrical indications, which could compromise both maternal and fetal nutrition and result in preterm delivery, low birth weight, and intrauterine growth restriction [5].

The clinical picture of PPD does not differ significantly from that of major depressive disorder (MDD) or from other depressive episodes that may appear during other periods of a woman's life [6]. However, it is the specific content of depressive ideation in the period following childbirth, with abnormal concern for the health of the baby and a decreased ability to face the common daily challenges of parenting, which explain why PPD is considered a disorder in its own right.

PPD can have severe repercussions on maternal and infant well-being and, in serious cases, may contribute to infant abuse, infanticide, and suicidal behaviour [1]. The risk of suicide is significantly elevated among depressed women during the perinatal period and has been found to be the second or leading cause of death in this depressed population. Self-harm ideation is more common than attempts or actual death, with thoughts of self-harm ranging from 5% to 14% [7].

Untreated postpartum affective illness is associated with significant long-term negative effects on child development and behaviour [8]. Since the infant's social environment consists primarily of the mother, and it is the mother who introduces the infant to the external world, PPD could affect the quality of the mother-child relationship and therefore the emotional, behavioural, and cognitive development of the newborn [9].

Maternal depression in the postnatal period has been associated with wide-ranging and persistent impairment in child functioning [10–12]. The most comprehensive study on the long-term effects of PPD on infants found that the children of PPD mothers performed significantly less well on cognitive tasks at 18 months of age than did the children of non-PPD mothers, particularly the boys [13]. Behavioural effects are variably supported and may persist up to 5 years of age postpartum and beyond. In particular, PPD may increase distractibility, antisocial or neurotic behaviours, and insecurity of attachment, all risk factors for the development of psychiatric disorders. Longitudinal studies have shown that the offspring of mothers suffering from depression during the postnatal period have a higher risk of developing affective disorder in adolescence, especially if there had also been later episodes of maternal depression [14].

Literature suggests a multifactorial etiology of PPD. Many studies have considered clinical (premature births, intrauterine growth impairment, operative deliveries, and admission to neonatal care units), biological (sleep disruption, deregulation of neurotransmitters, and serotonin), hormonal (thyroid, cortisol, and oxytocin), and psychological variables (stressful life events, marital conflict, and low social support, attachment insecurity, and personality features) as risk factor for PPD [15–17]. In particular, research into the pathophysiological mechanism underlying PPD has so far focused on hormones and their fluctuation [18]. However, a better understanding of the neural pathophysiology of PPD is a prerequisite for the effective prevention and treatment of this targeted disorder.

Magnetic resonance imaging (MRI) is increasingly used for its ability to noninvasively study the structure, ultrastructure, and functions of the brain in normal and pathological conditions. In mental disorders, particularly in MDD, MRI has already provided a wealth of potentially useful information that may, in the near future, help refine diagnosis and enable an improved prognosis beyond the current therapeutic response of this often treatment-resistant condition. The purpose of this review, following summarisation of studies which focus on different MR-based techniques used in MDD, is to identify and briefly describe those studies that deal specifically with PPD and, finally, to indicate the potential for future development of this positive line of investigation.

## 2. Achievements of MR-Based Research in MDD

In the last two decades many of the clinical instruments used to investigate the brain have been utilised in the study of MDD; these include electroencephalography [19], nuclear imaging techniques, that is, positron emission tomography (PET) and single photon emission computed tomography (SPECT) [20], X-ray computed tomography (CT), and MRI. The latter is the most commonly preferred technique for a variety of reasons: it is the most versatile; it does not emit ionizing radiation; it has the highest anatomical detail; it is widely available. This availability is due to the fact that it provides the reference morphostructural imaging modality used in diseases of the central nervous system.

The first MRI studies that focused on the brain morphometric correlates of patients with MDD appeared in the early 1990s, initially complementing and then rapidly replacing those conducted by CT. Several anatomical variations were noted between depressed and normal subjects, in particular the reduction of the volume of structures that have a critical role in the processing of emotions (prefrontal cortex, orbitofrontal cortex, cingulum, hippocampus, and striatum) and the presence of white matter abnormalities [21]. Studies of gross anatomy continue to provide intriguing results such as the recently reported higher prevalence of the right bending of the occipital lobe in MDD patients [22]. Newer techniques such as voxel-based morphometry and surface-based morphometry provide fine grain detail on several more subtle structural abnormalities of the cortex and subcortical structures in MDD [23–25]. Such MR-based quantitative assessments of neuroanatomical correlates of MDD offer new and clinically valuable insights into the neural mechanisms of the disease. Brain morphometric profiles may help discriminate milder MDD subtypes from those characterized by a more severe clinical picture. For example, hippocampal volumetric reductions have been shown to be more marked in patients with a higher number of depressive relapses and in those with a poorer response to antidepressants [26].

Diffusion tensor imaging (DTI) is an MRI technique used to investigate brain white matter microstructure. Measures of diffusion of molecular water such as fractional anisotropy and mean diffusivity are typically taken as indices of white matter health. By a distinct approach, various DTI-based tractography methods can also be used to probe the integrity of intra- and interhemispheric white matter pathways. In MDD patients, studies investigating structural connectivity found consistent abnormalities at the level of the frontolimbic circuits implicated in emotion and mood regulation, suggesting the presence of a dysconnection syndrome [27, 28]. On the other hand, the largest DTI study published so far failed to show a disruption in white matter integrity [29], suggesting that patients with white matter disruption may represent only a subgroup of MDD subjects. This finding is in line with the well-known clinical heterogeneity of MDD.

The main contribution of MRI to research the neural correlates of depression, however, is represented by the studies of activation utilising the technique “blood oxygen level dependent” (BOLD). With a latency of a few seconds following the administration of motor, sensory, or cognitive stimuli, BOLD MRI is able to depict specific local areas of brain hemodynamic response. The local increase of oxygen-rich blood flow causes in turn a rapid increase to the intensity of the MRI signal. The large amount of data resulting from activation studies with cognitive stimulation in mood disorders indicates the existence of a corticolimbic functional circuit. Its key structures are the medial prefrontal cortex and the amygdala, whose function is, respectively, decreased and increased in MDD compared with nondepressed state. Other dysfunctional circuits in MDD encompass paralimbic structures as the anterior cingulate cortex and subcortical structures as the thalamus [30].

The study of spontaneous BOLD signal fluctuations detectable in brains in the absence of specific stimuli is used increasingly to investigate functional alterations in brain circuits [31]. Resting-state functional connectivity studies are particularly suitable for use in patients with mental disorders who, in task-based activation studies, are often less cooperative than required [32]. The relatively small number of studies utilising resting-state functional connectivity in MDD provides results largely consistent with those of activation studies, revealing altered connections in the corticolimbic circuit and in the default mode network (DMN) [33, 34], a brain system that participates in internal modes of cognition.

Using MR spectroscopy, it is possible to study brain neurochemistry by measuring the level of relevant biochemical metabolites. The studies conducted into MDD were mainly carried out using proton MRS and assessed in particular the concentrations of *γ*-aminobutyric acid (GABA), glutamate, and the composite measure of glutamate and glutamine (Glx). The latter may be more sensitive than glutamate alone as an index of glutamatergic dysfunction in mental disorders [35]. What emerged with relative consistency were the reduced brain concentrations of GABA, glutamate, and Glx in depressed subjects, a finding that is more prominent in the occipital, prefrontal, and cingulate cortex [35–37]. The hypothesis that a glutamatergic deficiency represents a key neurochemical correlate of MDD has important therapeutic implications [38].

## 3. Literature Search of MR Studies in PPD

Papers were searched on MEDLINE, PsycINFO, Web of Science, and Scopus databases with the following key words: (“postpartum depression” or “postpartum mood disorder” or “perinatal depression” or “perinatal mood disorder”) AND (“neuroimaging” or “magnetic resonance imaging” or “magnetic resonance spectroscopy” or “diffusion tensor imaging” or “morphometry” or “connectivity” or “resting state”). We sought to identify additional studies by hand-searching bibliographies of retrieved articles and relevant reviews. The search was limited to papers published in English prior to December 2014. We did not include studies of healthy nondepressed mothers. Also excluded are reports of animal research.

## 4. Results

A total of 11 papers were identified, nine reporting on BOLD imaging studies (six activation studies and three resting-state functional connectivity studies) and two on MR spectroscopy studies. The salient features of these papers are summarized in Table 1.

### 4.1. Activation fMRI Studies

With their article published in 2007, Silverman et al. [39] were the pioneers of the use of brain MRI in the study of the neural correlates of PPD. Using fMRI the study focused on four women with PPD and four euthymic mothers that were subjected to a visual presentation task consisting of words with either a positive, negative, or neutral valence. The small sample was later increased to include six women with PPD and 11 euthymic mothers [40]. The analysis of data, obtained with the same paradigm, was aimed at the study of the amygdala. The authors found a reduced activation of the right amygdala after exposure to words with the meaning of threat, a pattern different to that usually observed in MDD.

Moses-Kolko et al. [41] have expanded these results using a task of exposure to negative facial expressions, showing an association between infant-related hostility and a reduced activation of the amygdala to the right. They have also documented that the dorsomedial prefrontal cortex is underactive and that the functional connectivity is decreased between these two structures. With fMRI the same group explored another dimension of the phenomenology of depression, the reaction to positive emotions that were found altered in MDD. Using a task of monetary reward these authors were able to show a rapid attenuation of striatal activation in depressed mothers [42].

Laurent and Ablow studied the reaction of depressed and nondepressed mothers to the sound of their own infant's cry compared to the cry of other infants and a noncontrol sound [43]. Their main finding was that while nondepressed mothers were activated in networks previously identified in normative parenting (limbic subcortical regions, dorsomedial prefrontal cortex, insula, and fusiform gyrus), the depressed group did not show any significant activation associated with the sound of their own infant's cry compared to either reference stimulus. In another series of experiments using the same sample above [44], the authors studied the mothers' reaction to their own infant's emotional facial expressions. Again, depressed mothers activated less, specifically in the dorsal anterior cingulate cortex when exposed to distressed facial expressions and in the orbitofrontal cortex and frontal insula when exposed to the expressions of joy. Of note, women participating in both studies were more than one year postpartum.

**Figure 1:**
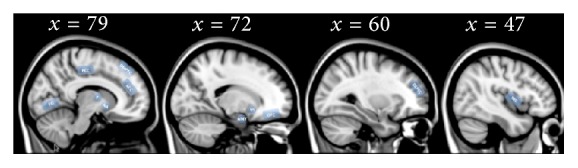
Overview of brain structures playing a role in the neurobiological mechanisms of postpartum depression as disclosed by BOLD activation fMRI studies [39–44]. Insula (INS), dorsolateral prefrontal cortex (DLPFC), dorsomedial prefrontal cortex (DMPFC), orbitofrontal cortex (OFC), thalamus (T), nucleus accumbens (NA), amygdala (AMY), ventral striatum (VS), fusiform gyrus (FG), anterior cingulate cortex (ACC), and posterior cingulate cortex (PCC). A template structural image provided with FSL software MNI 152 1 mm was used for anatomical reference. MNI coordinates of the selected slices are also indicated.

Figure 1 provides a pictorial review of the main areas showing activation changes in women with PPD compared to control subjects in the studies above.

### 4.2. Resting-State Functional MRI Studies

Three studies investigated functional brain connectivity in depressed mothers using resting-state MRI. Deligiannidis et al. [45] found that, compared to healthy mothers, PPD subjects had a reduced connectivity for anterior cingulate cortex, amygdala, hippocampus, and dorsolateral prefrontal cortex and between the corticocortical and corticolimbic regions.

Building on the results of the activation studies carried out by their own group [41], Chase et al. [46] chose to investigate functional connectivity in the DMN regions known to be involved in social cognition in PPD. Disrupted connectivity was found between the posterior cingulate cortex (a key structure in the DMN) and the right amygdala in depressed subjects compared to nondepressed, healthy mothers.

A third resting-state study [47] focused on local connectivity by investigating changes in regional homogeneity (ReHo), an approach distinct from the mainstream strategy of studying the temporal correlation of low frequency fluctuations between remote brain regions. The authors found in depressed mothers an altered (either increased or decreased) regional homogeneity in the posterior cingulate cortex, medial frontal cortex, and temporal cortex.

### 4.3. MR Spectroscopy Studies

Our search identified only two studies conducted using MR spectroscopy in PPD. Overall only 21 PPD patients were studied. Epperson et al. [48] assessed brain GABA concentration using a J-edited sequence and a single voxel placed on the medial occipital lobes. Compared to a reference group of healthy follicular-phase women, cortical GABA was reduced in postpartum women, regardless of their mood. In a MRS assessment of glutamate levels in the medial prefrontal cortex using a stimulated echo acquisition mode (STEAM) sequence, McEwen et al. [49] found a slightly increased glutamate concentration, at variance with the observed reduction in the majority of MRS studies in MDD [38].

### 4.4. Other MRI Studies

With the methodology adopted we were unable to find in published research any study where MRI was used to investigate possible brain abnormalities in women with PPD using techniques other than those cited above. In particular, there were no studies using voxel-based morphometry, surface-based morphometry, and diffusion-tensor imaging.

## 5. Discussion

The major result of our search is the discovery that there is a dearth of published studies investigating the neurobiology of PPD using MR techniques. A concurrent search conducted using key words such as “major depression,” “major depressive disorder,” and “magnetic resonance” in various combinations yielded more than 1000 unselected entries. The reason for this shortage of contributions is easy to imagine. Despite the high prevalence of PPD, recruiting both normal and depressed women in MRI studies is particularly difficult shortly after delivery. Overall, brain MRI findings in PPD appear to replicate those obtained in MDD. Two exceptions, a reduced activation of the right amygdala after exposure to words with the meaning of threat [40], rather than the hyperactivity observed in comparable MDD studies, and a slight increase in spectroscopy-detected glutamate concentration in the frontal cortex [49], which contrasts with an opposite finding in most MRS studies in MDD, are not sufficient to delineate a distinct neurobiological profile for PPD, due to the small samples used to make these assessments and the lack of direct comparisons with MDD subjects. However, it seems reasonable to expect that MR studies conducted in larger populations of mothers with PPD might allow for the identification of MRI signatures for this condition.

One of the significant features of PPD is the impact mood disorders have on the mother-child relationship. This area of interest suggests that children of a mother who has had postnatal depression are more likely to have cognitive and emotional problems throughout development. Depressed mothers, in fact, gaze less at their infants, rock their infants less, are less active and decisive, have less well-timed responsiveness, demonstrate lower levels of warm acceptance, and are emotionally flat and often disengaged [12, 50–52]. Infant development is powerfully shaped by the quality of the early mother-infant interaction following PND [53, 54]. In recent years, several instruments have been developed to assist clinicians in detecting disorders in the early emotional bonding between a mother and her child [55–57].

Of the few studies conducted with neuroimaging, the first attempt to study the neural correlates of mother/child interaction with MRI was reported by Lorberbaum et al. [58]. These authors used fMRI to compare the difference in the reactions of mothers to a baby's cry with those to noise or nonspecific auditory stimuli. The results showed the activation of a circuit integrating the cingulate cortex, the medial thalamus, the medial prefrontal cortex, and the orbitofrontal cortex, congruous with the results of animal research. Most of the studies published to date have used MRI to identify brain alterations in depressed mothers exposed to emotional stimuli and have documented several deviations from the activation patterns typical of normal motherhood. As pointed out in a recent critical review of these results, along with the results of other studies conducted using fMRI on both normal and depressed mothers, it will be necessary to build on existing data using more homogeneous clinical phenotypes, more refined experimental paradigms, and larger samples [59].

Aside from activation studies, MRI offers an unparalleled set of noninvasive techniques that can shed light on the neural correlates of mental disorders. It is time for researchers in the field of PPD to utilise the potential of MRI and to eradicate the gap with conditions such as MDD, schizophrenia, and obsessive-compulsive disorder that have already benefitted from the diagnostic ability of brain MR. The classification of mental disorders is in transition from the traditional nosological categories to a more patient-centred classification based on prognosis and response to specific treatments [26]. MRI can contribute significantly to the establishment of this new classification system and PPD should be no exception to this trend. Studies using resting-state functional connectivity, voxel- and surface-based morphometry, DTI, and spectroscopy that for the time being are absent in the published literature are poised to be adopted in PPD research as they are, in general, easier for patients to undergo and more inclined to standardisation than activation studies.

## Figures and Tables

**Table 1:** 

Silverman et al., 2007 [39]	BOLD activation (pilot study)	*N* = 8 (4 PPD) 4–6 weeks from delivery	Exposure to emotionally valenced stimuli: ↓ activation of R amygdala

Silverman et al., 2011 [40]	BOLD activation	*N* = 17 (6 PPD) 4–6 weeks from delivery	Same as above

Moses-Kolko et al., 2010 [41]	BOLD activation	*N* = 20 (14 PPD) within 12 weeks from delivery	Exposure to faces and shapes: ↓ L dorsomedial prefrontal face-related activity, ↓ L amygdala activity in more severe PPD

Moses-Kolko et al., 2011 [42]	BOLD activation	*N* = 24 (12 PPD) avg. 8 weeks from delivery	Monetary reward guessing task: rapid attenuation of ventral striatal activity

Laurent and Ablow, 2012 [43]	BOLD activation	*N* = 22 (11 PPD) 15–18 months from delivery	Exposure to own infant's cry, to other infants' cry, and to control sound. In depressed mothers no activation for own infant versus control plus ↓ activation in caudate, accumbens, and medial thalamic. In less depressed mothers ↑ act in L orbitofrontal, dorsal anterior cingular cortex, and medial superior frontal. Only in nondepressed mothers, activation in fusiform gyrus (own versus other infants)

Laurent and Ablow, 2013 [44]	BOLD activation	*N* = 22 (11 PPD) 15–18 months from delivery	Exposure to own infant's distress faces: in more depressed mothers, ↓ activation of dorsal anterior cingulate cortex. Exposure to own infant's joy faces: in depressed mothers, ↓ orbitofrontal and insular activity. Exposure to joy-distress faces reduced L prefrontal and insular striatal act

Wang et al., 2011 [47]	BOLD resting state	*N* = 21 (10 PPD) within 16 weeks of delivery	↑ activity consistency in cingulate cortex and frontal and parietal lobe ↓ activity consistency in temporal and frontal lobe (regional homogeneity (ReHo) analysis)

Deligiannidis et al., 2013 [45]	BOLD resting state	*N* = 17 (9 PPD) within 9 weeks of delivery	↓ connectivity with anterior cingulate cortex, amygdalae, hippocampi, and dorsolateral prefrontal cortex

Chase et al., 2014 [46]	BOLD resting state	*N* = 37 (14 PPD) avg. 8 weeks from delivery	↓ posterior cingular cortex connectivity with R amygdala

Epperson et al., 2006 [48]	MR spectroscopy	*N* = 35 (9 PPD) within 24 weeks from delivery	Trend to ↓ GABA levels in occipital cortex

McEwen et al., 2012 [49]	MR spectroscopy	*N* = 24 (12 PPD) within 12 weeks from delivery	↑ glutamate levels in medial prefrontal cortex
